# Prognostic value of serum brain-derived neurotrophic factor levels in patients with Chagas cardiomyopathy

**DOI:** 10.1590/0074-02760180224

**Published:** 2018-08-20

**Authors:** Henrique S Costa, Marcia Maria O Lima, Pedro Henrique S Figueiredo, Patrícia M Martinelli, Elizabeth RS Camargos, Ana Thereza Chaves, Maria Carmo Pereira Nunes, Manoel Otavio C Rocha

**Affiliations:** 1 Universidade Federal de Minas Gerais, Departamento de Clínica Médica, Curso de Pós-Graduação em Infectologia e Medicina Tropical, Belo Horizonte, MG, Brasil; 2 Universidade Federal dos Vales do Jequitinhonha e Mucuri, Faculdade de Ciências Biológicas e da Saúde, Departamento de Fisioterapia, Diamantina, MG, Brasil; 3 Universidade Federal de Minas Gerais, Instituto de Ciências Biológicas, Departamento de Morfologia, Belo Horizonte, MG, Brasil

**Keywords:** brain-derived neurotrophic factor, Chagas cardiomyopathy, echocardiogram, exercise testing, prognosis

## Abstract

**BACKGROUND:**

Serum brain-derived neurotrophic factor (BDNF) levels have been shown to be lower in patients with Chagas cardiomyopathy (ChC) than in patients with non-dilated chagasic cardiomyopathy. However, its prognostic value was not established in patients with ChC.

**METHODS:**

Forty-nine patients with ChC (50 ± 7 years, New York Heart Association “NYHA” I-III); were evaluated by echocardiography, exercise testing, and blood analysis. Serum BDNF levels were determined using enzyme-linked immunosorbent assay sandwich. Patients were followed-up, and cardiac death was considered the end-point. The survival analyses were performed using Kaplan-Meier and Cox regression.

**RESULTS:**

After 39 ± 14 months of follow-up, 12 patients (25%) died. The concentration of 2.5 ng/mL was the optimal cut-off value to predict survival with significant difference between the groups with low (≤ 2.5 ng/mL) and high (> 2.5 ng/mL) BDNF levels (p = 0.006). Lower serum BDNF levels (hazards ratio (HR) 1.1, 95% confidence interval (CI) 1.1-1.4; p = 0.001), peak oxygen uptake (HR 1.2, 95% CI 1.0-1.3; p = 0.009), and left ventricular ejection fraction (HR 0.8, 95% CI 0.7-0.9; p = 0.001) were the independent predictors of survival. The combination of low serum BDNF levels and reduced left ventricular ejection fraction were highly predictive of death (HR 5.6, 95% CI: 1.2-9.7; p = 0.026).

**CONCLUSION:**

In patients with ChC, reduced serum BDNF levels, especially if associated with systolic function, may provide useful prognostic information.

Chagas disease still remains an important public health problem in Latin America.^1^ Due to immigration and globalisation, its prevalence has increased in the last decades both, in Europe^2^ and the United States.^3^ Dysautonomia^4^, inflammatory cytokine expression^5^, and functional impairment^6^ are important clinical features in all stages of infection, especially in Chagas cardiomyopathy (ChC), the most severe clinical manifestation of the disease.

Brain-derived neurotrophic factor (BDNF) is a neurotrophin widely distributed in the central nervous system^7^ that regulates various neurotrophic functions^8^ and is involved in metabolic^9^ and inflammatory^10^ processes. In patients with heart diseases, BDNF has proved to be a valuable cardioprotective factor against ischemic injury after myocardial infarction,^11^ and low BDNF levels were associated with worse prognosis in patients with heart failure and angina pectoris.^12^

A previous study^13^ showed that serum BDNF levels are lower in patients with ChC than in those with non-dilated chagasic cardiomyopathy (p < 0.05) by intense fibrosis. Furthermore, other studies have reported the positive effect of moderate aerobic exercise after a single exercise session^14^ and after exercise training^15^ on serum BDNF levels. However, the role of serum BDNF levels on the survival of patients with ChC remained unknown. The present study aimed to verify the prognostic value of serum BDNF levels in patients with ChC.

## SUBJECTS AND METHODS

A prospective study with clinically stable patients with ChC was conducted at scheduled clinic visits from an Outpatient Reference Centre for Chagas Disease in the state of Minas Gerais, Brazil. The research was approved by the Institutional Ethics Committee, and all patients gave their written informed consent before participating.

A minimum of two positive serologic tests for antibodies against *Trypanosoma cruzi* were required for the diagnosis of Chagas disease. To be included in the present study, patients should also have clinical and electrocardiographic findings compatible with ChC^16^ and dilated left ventricle with impaired left ventricular systolic function (left ventricular ejection fraction ≤ 45%). Exclusion criteria were the presence of systemic or heart disease by any other causes or comorbidities, blood transfusion within six months, use of antidepressant medication, and inability to perform exercise test.

At baseline, the blood sample was collected, and previous patients underwent echocardiogram and symptom-limited exercise testing.

*Blood sample and serum BDNF analysis* - A 5-mL sample of blood was collected by venepuncture using a sterile Vacuntainer flask without anticoagulant, after patients were rested for 30 min. Serum samples were stored at -80ºC, and BDNF levels were determined by enzyme-linked immunosorbent assay (ELISA),^17^ according to the R&D Systems protocol (Minneapolis, MN, USA). The biochemical analysis was performed by two different researchers.

*Echocardiography evaluation* - Left ventricular ejection fraction (LVEF) was obtained using modified Simpson’s rule. Diastolic function was assessed using pulsed-wave Doppler examination of mitral inflow and tissue Doppler imaging (TDI). Early diastolic velocity (e’) at the medial border of the mitral annulus was obtained, and the ratio between peak mitral E and e’ (E/e’) was calculated.

*Treadmill exercise testing* - A symptom-limited exercise test was performed on a treadmill (Digistress Pulsar, Micromed, Brazil) using a standard Bruce protocol. On the day of the treadmill test, patients received their usual cardiac medications and were requested to abstain from eating, drinking, smoking, and performing rigorous physical activity for at least 3 h before the test. A 12-lead electrocardiogram was continuously monitored and recorded every 1 min. The maximal exercise capacity was verified by VO_2peak_, expressed in mL/kg/min and calculated indirectly using the formula VO_2peak_ = 2.33 (time in min) + 9.48.^18^

*Follow-up period* - Follow-up started after the baseline evaluations and was conducted through scripted telephone interviews every four months for five years. The end-point was defined as cardiac death.

*Statistical analysis* - The data distribution was verified by the Kolmogorov-Smirnov test. The descriptive analysis was expressed as the mean with standard deviation or median and interquartile range, as appropriate. Categorical variables are presented as absolute number (percentage). Independent T-test, chi-square, and Mann-Whitney were performed for data analysis, with significance levels at 0.05.

The prognostic value of BDNF levels was verified with uni- and multivariate Cox regression analysis. In the Cox regression model, sex, New York Heart Association (NYHA) functional class, and BDNF levels (cut-off value) were used as categorical variable. The other variables were continuous.

A receiver operating curve was obtained to determine the cut-off value of the serum BDNF levels and variables that remained as independent predictors of cardiac death in the multivariate Cox analysis. The optimal cut-off considered was the value with the best combination of sensitivity and specificity to predict cardiac death. The cut-off value was used in the Kaplan-Meier curve.

Data were analysed with SPSS software, version 20.0 (Chicago, Illinois).

## RESULTS

A total of 49 patients with ChC were evaluated. The median serum BDNF concentration was 6.2 (1.9-8.5) ng/mL. Serum BDNF levels, demographic data, functional status, and echocardiographic features are listed in Table I.

By the final follow-up (39 ± 14 months), 12 patients (25%) had died. Non-survivors had lower serum BDNF levels (p = 0.030) and lower VO_2peak_ compared to survivors. Inter-group differences are shown in Table II.

**Fig. 1:**
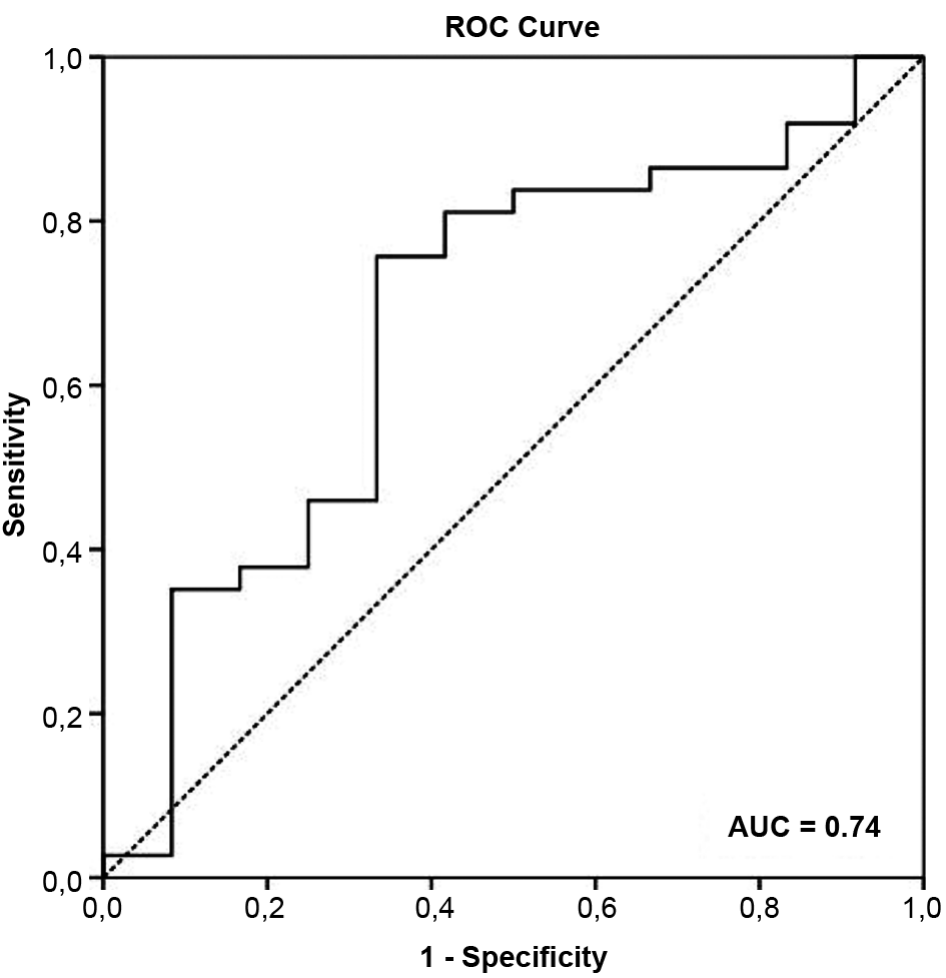
accuracy of serum brain-derived neurotrophic factor (BDNF) levels in predicting cardiac death based on the receiver operating characteristic (ROC) curve in patients with Chagas cardiomyopathy (ChC).

The area under the receiver operating characteristic (ROC) curve to identify the risk of cardiac death according to serum BDNF levels in patients with ChC was 0.74 (95% CI: 0.56-0.93) (Fig. 1) and the concentration of 2.5 ng/mL was the optimal cut point value, with 75% sensitivity and 70% specificity. Based on this cut-off point, the groups were stratified into low-BDNF level group (17 patients with serum BDNF levels of ≤ 2.5 ng/mL) and high-BDNF level group (32 patients with serum BDNF levels of > 2.5 ng/mL).

The frequency of cardiac death was higher in the low-BDNF level group (≤ 2.5 ng/mL) than in the high-BDNF level group (> 2.5 ng/mL; 47% versus 12%; p = 0.011).

**TABLE I:** Baseline characteristics of the sample

Variables	All patients (n = 49)
Serum BDNF (ng/mL)	6.2 (1.9 - 8.5)
Age (years)	50 ± 7
Male sex (%)	28 (57)
BMI (kg/m^2^)	24.8 ± 4.0
NYHA class (%)	
I	28 (57)
II	16 (33)
III	5 (10)
Medication, n (%)	
Amiodarone	29 (59)
β-blockers	15 (31)
ACE-inhibitor	34 (71)
Diuretics	35 (71)
Digitalis	12 (24)
Anticoagulants	06 (12)
Exercise testing	
VO_2peak_ (mL.kg.min)	27.2 ± 7.4
Echocardiography	
LVEF (%)	36.0 (31.0 - 41.0)
LVDd (mm)	63.5 ± 6.3
E/e’ ratio	10.9 ± 5.0

Data presented as mean and standard deviation (mean ± SD), median (MD) and interquartile range (25-75%) or absolute number (percentage). BDNF: brain-derived neurotrophic factor; BMI: body mass index; E/e’ ratio: ratio of the early diastolic transmitral flow velocity to early diastolic mitral annular velocity; LVDd: left ventricular end-diastolic diameter; LVEF: left ventricular ejection fraction; ng/mL: nanograms per milliliters; NYHA: New York Heart Association functional class; VO2peak: peak oxygen uptake.

**TABLE II:** Differences in serum brain-derived neurotrophic factor (BDNF), demographic data, functional status and echocardiographic parameters between survivors and non-survivors patients

Variables	Survivors (n = 37)	Non-survivors (n = 12)	p-value
Serum BDNF (ng/mL)	4.4 (2.4 - 11.9)	2.0 (1.4 - 5.2)	0.041
Age (years)	50 ± 7	49 ± 9	0.926
Sex (male/female)	20/17	8/4	0.336
NYHA class I/II/III	23/12/2	5/4/3	0.391
Exercise testing			
VO_2peak_ (mL.kg.min)	30.7 ± 7.9	26.1 ± 6.9	0.048
Echocardiography			
LVEF (%)	37.0 (32.5 - 41.0)	32.0 (24.2 - 40.5)	0.169
LVDd (mm)	65.2 ± 6.5	65.8 ± 5.8	0.449
E/e’ ratio	10.5 ± 5.5	11.0 ± 4.9	0.651

Data presented as mean and standard deviation (mean ± SD), median (MD) and interquartile range (25-75%) or absolute number. p-values highlighted in bold are statistically significant (p < 0.05). BMI: body mass index; E/e’ ratio: ratio of the early diastolic transmitral flow velocity to early diastolic mitral annular velocity; LVDd: left ventricular end-diastolic diameter; LVEF: left ventricular ejection fraction; ng/mL: nanograms per milliliters; NYHA: New York Heart Association functional class; VO_2peak_: peak oxygen uptake.

The univariate Cox analysis showed that lower serum BDNF levels, reduced VO_2peak_, and lower LVEF were associated with poor prognosis at the end of follow-up (Table III). In the final multivariate model, lower BDNF levels [hazards ratio (HR) 1.1, 95% confidence interval (CI): 1.1-1.4; p = 0.001], LVEF (HR 0.8, 95% CI: 0.7-0.9; p = 0.001), and VO_2peak_ (HR 1.2, 95% CI: 1.0-1.3; p = 0.009) remained as independent predictors of cardiac death in patients with ChC.

When comparing the variables as independent predictors of cardiac death using the Cox regression analysis, the serum BDNF level showed a prognostic value similar to that of LVEF, based on the area under the ROC curve and negative and positive predictive values (Table IV). The prognostic value of VO_2peak_ was lower than that of serum BDNF and LVEF.

**Fig. 2:**
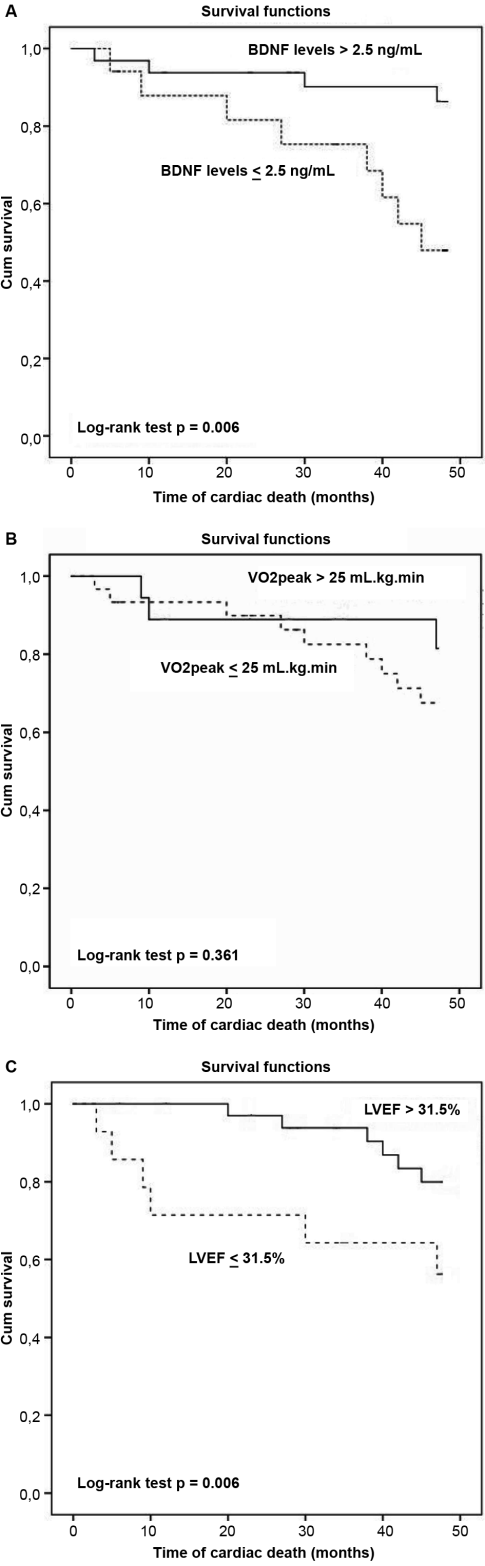
Kaplan-Meier curve for (A) serum brain-derived neurotrophic factor (BDNF) levels, (B) VO_2peak_, and (C) left ventricular ejection fraction (LVEF). The cut-off points were determined using the receiver operating characteristic (ROC) curve.

In the Kaplan-Meier analysis using the cut-off points obtained using the ROC curve, only VO_2peak_ showed no significant difference (log rank = 0.361) between the groups with values below and above the established point, that is, 25 mL/kg/min (Fig. 2).

In Cox survival analysis using the cut-off points obtained with the ROC curve, the combination of low BDNF values (≤ 2.5 ng/mL) and LVEF (≤ 31.5%) were highly predictive of cardiac death (HR 5.6, 95% CI: 1.2-9.7; p = 0.026).

## DISCUSSION

The present study showed, for the first time, the prognostic value of serum BDNF levels in patients with ChC. The main findings of the present study were as follows: (1) serum BDNF levels and VO_2peak_ were lower in non-survivor patients with ChC than in the survivors; (2) serum BDNF concentration of 2.5 ng/mL was the optimal cut-off value to identify patients at risk of adverse outcome; (3) low serum BDNF levels, LVEF, and VO_2peak_ were independent predictors of cardiac death, and (4) the combination of low serum BDNF levels and LVEF are highly predictive of cardiac death in ChC after 39 ± 14 months of follow-up. Our results suggest that serum BDNF levels, especially when associated with systolic function, may provide useful prognostic information in patients with ChC.

Three studies^19^ ^-^ ^21^ reported the efficacy of circulating BDNF levels in predicting adverse events in patients with heart failure,. Fukushima et al.^19^ evaluated 58 patients (59.2 ± 13.7 years, NYHA I-III) and found that after 20.3 months of follow-up, BDNF and brain natriuretic peptide (BNP) levels were independent predictors of adverse events in patients with heart failure. Low BDNF levels (< 17.4 ng/mL) were more significantly associated with morbidity and mortality than high BDNF levels (≥ 17.4 ng/mL) (HR 0.41, 95% CI 0.20-0.84; p = 0.003). Similarly, Kadowaki et al.^20^ evaluated a large number of patients with heart failure (n = 134, 71 ± 13 years, NYHA II-IV) and demonstrated that low BDNF levels (≤ 12.4 ng/mL) had poor prognosis compared to high BDNF concentration (> 12.4 ng/mL) (log rank test: p = 0.0005). Low BDNF levels were independently associated with cardiovascular adverse outcomes (HR 2.93, 95% CI: 1.62-5.30; p < 0.001), as well as BNP, age, and estimated glomerular filtration rate.

**TABLE III:** Uni and multivariate Cox analysis for cardiac death in Chagas cardiomyopathy (ChC) patients

Variables	Univariate				Multivariate		
	HR	95% CI	p-value		HR	95% CI	p-value
Lower BDNF levels (≤ 2.5 ng/mL)	1.2	1.1 - 1.7	0.013		1.1	1.1 - 1.4	0.001
Age (years)	0.9	0.9 - 1.1	0.961		-	-	-
Sex (male *vs* female)	0.6	0.2 - 2.0	0.425		-	-	-
NYHA functional class II and III	1.0	0.2 - 1.6	0.597		-	-	-
VO_2peak_ (mL.kg.min)	1.1	1.0 - 1.2	0.038		1.2	1.0 - 1.3	0.009
LVEF (%)	0.9	0.9 - 1.0	0.046		0.8	0.7 - 0.9	0.001
LVDd (mm)	1.0	0.9 - 1.1	0.763		-	-	-
E/e’ ratio	1.0	0.9 - 1.1	0.871		-	-	-

BDNF: brain-derived neurotrophic factor; E/e’ ratio: ratio of the early diastolic transmitral flow velocity to early diastolic mitral annular velocity; HR: hazard ratio; LVDd: left ventricular end-diastolic diameter; LVEF: left ventricular ejection fraction; ng/mL: nanograms per milliliters; NYHA: New York Heart Association functional class; VO2peak: peak oxygen uptake; 95% CI: 95% confidence interval.

**TABLE IV:** Cutoff values, area under the receiver operating characteristic (ROC) curve, sensitivity, specificity and positive and negative predictive values of independent predictors of cardiac death

Predictors	Cutoff value	AUC (95% CI)	Sensitivity	Specificity	Negative predictive values	Positive predictive values
Serum BDNF	2.5 ng/mL	0.74 (0.56-0.93)	75%	70%	0.31	0.76
VO2_peak_	25 mL.kg.min	0.69 (0.50-0.90)	75%	60%	0.17	0.59
LVEF	31.5%	0.76 (0.59-0.95)	80%	73%	0.43	0.78

AUC: area under the curve; BDNF: brain-derived neurotrophic factor; ng/mL: nanograms per milliliters; LVEF: left ventricular ejection fraction; VO2peak: peak oxygen uptake; 95% CI: 95% confidence interval.

Takashio et al.^21^ reported that plasma concentration of BDNF in patients with heart failure (n = 242, 71 ± 12 years, NYHA I-III) was significantly lower than in healthy individuals (p < 0.001). At the end of follow-up, 14% of patients died of any cause. Patients with low plasma BDNF concentration (≤ 3.7 ng/mL) had higher mortality rate than those with higher plasma BDNF levels (HR 2.22, 95% CI: 1.03-4.82, p = 0.04).

In the present study, lower BDNF level was an independent predictor of death in patients with ChC. Abnormalities in the skeletal muscles, autonomic denervation, and reduction of cardiomyocytes by fibrosis, the main pathological signs of an infected heart, are common clinical findings with the progression of Chagas disease. Both skeletal^22^ and cardiac muscles^23^ are important sources of BDNF. In addition, we believe that BDNF, being a neurotrophic factor, may reflect the autonomic dysfunction presented by patients with ChC. Thus, muscle atrophy, myocardial fibrosis, and autonomic denervation, the signs of disease progression, may contribute to the reduction of circulating levels of this neurotrophic factor.

The association between BDNF levels and predictors of poor prognosis in ChC has also been reported in another study^13^, which found a positive correlation between serum BDNF and LVEF levels (r = 0.3137, p = 0.0431) and a negative correlation with ventricular dilatation index (r = -0.3146, p = 0.0424). In patients with heart failure, a previous study^19^ reported that compared to BNP, the first-line biomarker in the prognosis of this disease, the BDNF was similarly effective in predicting adverse events in this population (AUC 0.827 and 0.798, respectively).

However, compared to the aforementioned studies, the present study showed different HR values. A few hypotheses are proposed. Firstly, Kadowaki et al.^20^ and Fukushima et al.^19^ considered cardiac death and hospitalisations due to heart failure as end-points, while the present study considered only cardiac death as the end-point. Secondly, studies by Kadowaki et al.^20^ and Fukushima et al.^19^ selected older patients (71 ± 13 and 59.2 ± 13.7 years, respectively) than those enrolled in the present study (50 ± 7 years). Furthermore, both studies used a sample set of patients with heart failure and comorbidities, such as diabetes, dyslipidaemia, and hypertension, which have been shown to reduce patient survival. Finally, the study by Kadowaki et al.^20^ did not report the use of antidepressant medication as an exclusion criterion; such medication could alter BDNF levels, reduce potential depressive symptoms, increase patient’s quality of life, and increase adherence to treatment.

The cut-off point of serum BDNF in the prediction of cardiac death was also found to be different in the present study compared to those in the studies by Fukushima et al.^19^ and Kadowaki et al.^20^. ChC evolves with incessant and progressive fibrosis. Moreover, fibrosis causes destruction of cardiomyocytes, which are important sources of BDNF, as justified by Martinelli et al.^13^. Thus, the baseline concentration of serum BDNF and the cut-off point in predicting cardiac death were expected to be lower in patients with heart failure due to ChC than in other aetiologies. Corroborating with this hypothesis, BDNF levels at baseline were lower in the present study [6.2 (1.9-8.5) ng/mL] when compared to the study by Fukushima et al.^19^ (19.0 ± 5.6) and Kadowaki et al.^20^ (14.7 ± 8.4 ng/mL).

Although low concentrations of serum BDNF are a statistically significant prognostic marker in ChC, it demonstrates a borderline HR when analysed alone. However, when combined with reduced LVEF, a well-established independent predictor of death in ChC, HR has been demonstrated to become clinically relevant. Patients with BDNF concentration of below 2.5 ng/mL and LVEF of < 31.5% had HR of 5.6 (95% CI: 1.2-9.7; p = 0.026) for cardiac death. Therefore, the association between reduced serum BDNF levels and LVEF had a higher prognostic value (HR 5.6, 95% CI: 1.2-9.7) than LVEF alone (HR 0.8, 95% CI: 0.7-0.9), which may have an impact on the assessment of prognosis and risk stratification of the patient. We believe that patients with serum BDNF level of below 2.5 ng/mL and LVEF of below 31.5% are high risk and should be monitored more frequently in the clinical management. In addition, patients with low serum BDNF and reduced LVEF levels should be given priority in exercise-based treatment, as physical training in ChC appears to increase the BDNF levels.^15^

In the multivariate Cox analysis, LVEF and VO_2peak_, as well as the low concentration of serum BDNF level, remained as independent predictors of death. The LVEF is a well-established independent predictor of survival in patients with ChC^24^ ^,^ ^25^ and serum BDNF showed similar predictive values. However, the role of VO_2peak_ in predicting adverse events in this population remains poorly understood. Mady et al.^26^ conducted one of the first studies that aimed to verify the prediction of adverse events in male patients with ChC (n = 104, 40.3 ± 9 years) based on VO_2peak_. Under Cox multivariate regression, VO_2peak_ (p < 0.001) and LVEF (p < 0.001) were highly associated with survival time. Functional impairment has also been shown to suggest subclinical myocardial injury, preceding even the reduction of LVEF.^6^ Additionally, Ritt et al.^27^ found that VO_2peak_ was associated with poor prognosis in patients with ChC (n = 55, LVEF < 45%), but was not considered as an independent predictor of death (HR: 0.97, 95% CI: 0.91-1.04, p = 0.44). In the present study, VO_2peak_ was observed to demonstrate a significant predictor of survival, but failed to determine the cut-off point for the stratification of patients at higher risk of cardiac death. We believe that functional capacity is associated with patient’s clinical condition; however, the establishment of the role of VO_2peak_ on survival should be better investigated in studies with larger sample size.

Study limitations include small sample size and the performance of the stress test using conventional maximal exercise testing, without direct gas analysis. However, our sample size is in agreement with a prognostic study of BDNF in patients with heart failure.^19^ Regarding exercise testing, the indirect assessment of the VO_2peak_ has been established to be highly correlated with the direct measurement,^28^ ^)^ which does not compromise our results.

In conclusion, low serum BDNF level, especially in association with systolic function and functional capacity, was an independent predictor of survival in patients with ChC and may aid in the risk stratification of these patients.
